# ChatGPT for digital pathology research

**DOI:** 10.1016/S2589-7500(24)00114-6

**Published:** 2024-07-09

**Authors:** Mohamed Omar, Varun Ullanat, Massimo Loda, Luigi Marchionni, Renato Umeton

**Affiliations:** Department of Pathology and Laboratory Medicine, Weill Cornell Medicine, New York, NY, USA (M Omar MD, M Loda MD, L Marchionni MD, R Umeton PhD); Department of Informatics & Analytics, Dana Farber Cancer Institute, Boston, MA, USA (V Ullanat MS, M Loda, L Marchionni, R Umeton)

## Abstract

The rapid evolution of generative artificial intelligence (AI) models including OpenAI’s ChatGPT signals a promising era for medical research. In this Viewpoint, we explore the integration and challenges of large language models (LLMs) in digital pathology, a rapidly evolving domain demanding intricate contextual understanding. The restricted domain-specific efficiency of LLMs necessitates the advent of tailored AI tools, as illustrated by advancements seen in the last few years including FrugalGPT and BioBERT. Our initiative in digital pathology emphasises the potential of domain-specific AI tools, where a curated literature database coupled with a user-interactive web application facilitates precise, referenced information retrieval. Motivated by the success of this initiative, we discuss how domain-specific approaches substantially minimise the risk of inaccurate responses, enhancing the reliability and accuracy of information extraction. We also highlight the broader implications of such tools, particularly in streamlining access to scientific research and democratising access to computational pathology techniques for scientists with little coding experience. This Viewpoint calls for an enhanced integration of domain-specific text-generation AI tools in academic settings to facilitate continuous learning and adaptation to the dynamically evolving landscape of medical research.

## Introduction

In the last two decades, the fields of data science and artificial intelligence (AI) have heralded a new era in the way we analyse and interpret vast volumes of data. These innovations, made possible by powerful algorithms and vast computational resources, have permeated numerous sectors, ranging from finance and marketing to health care and scientific research. Amid this growing AI landscape, a particular subset of models known as large language models (LLMs) began to draw attention for their extensive capabilities in handling textual information. Although these models were known mostly within AI circles and some industries, the introduction of OpenAI’s CahatGPT on Nov 30, 2022, drew a broader audience, illustrating not just the capabilities of one LLM but also hinting at the untapped potential of these models in a multitude of applications. As we progress, this Viewpoint will delve deeper into the nuances of LLMs and the challenges they might present, especially when applied to niche scientific domains requiring specialised knowledge. We will also explore how domain-specific text-generative AI tools could be pivotal in addressing the limitations of LLMs, offering enhanced accuracy and precise responses in specialised areas of scientific disciplines.

## LLMs: bridging knowledge gaps

Building on the momentum of AI’s rapid evolution, the advent of LLMs has redefined the landscape of natural language processing. Models including generative pre-trained transformer (GPT)-3, GPT-4, and others (table 1) stand out for their vast parameters and ability to process large amounts of textual data in applications ranging from text generation to complex question answering, owing to LLMs’ intricate architecture and comprehensive training datasets.

In medical research, a key use case for LLMs lies in their potential to bridge the growing gap between the influx of new scientific results and the capability of researchers and institutions to stay updated about new information. This issue is particularly pivotal in fields where the rapid assimilation of new knowledge is crucial, and both stakes and risks are high. While only very few multimodal foundation models (eg, PathChat from Modella AI, and GigaPath from Microsoft Research) are ready to support end-to-end digital pathology research workflows, for instance, more text-based foundation models (ie, LLMs) can assist researchers by aggregating and summarising relevant information from existing literature, thereby aiding in hypothesis generation and knowledge discovery. For many in the scientific community, tools, such as ChatGPT, promised an era in which AI can augment human intellect to accelerate scientific discoveries or at least streamline knowledge retrieval and summarisation from the literature. This potential fusion of AI with human expertise raises the question: are we on the brink of a transformative era in medical research in which swift and easy access to comprehensive information is the standard, not the exception?

## Domain-specific challenges of LLMs

Although the capabilities of LLMs are undeniably impressive, their adaptability is often questioned when applied to specialised disciplines. These niche fields, due to their depth and intricacy, demand an unparalleled level of precision and comprehension. A general-purpose LLM, regardless of its vast training data, might not be fine-tuned enough to capture the nuanced details that human specialists rely on. Instead, there is a propensity for these models to default to generic, high-level responses—information that, while technically accurate, might not have sufficient specialised focus and depth essential for expert tasks.^10^

Taking digital pathology as a case study underscores these challenges further. Digital pathology is a relatively new sub-field of pathology that involves capturing and analysing histopathology images generated from glass slides with scanners.^11^ In this rapidly evolving field, expanding datasets, novel algorithms, and innovative applications aimed at streamlining clinical integration are continuously produced. As a result, keeping pace with this rapid influx of knowledge is challenging for practitioners who must sift through an ever-growing body of literature. In this context, the one-size-fits-all answers of LLMs fall short, missing the specificity necessary to support the nuanced decision making that this field demands.

## Advancements in domain-specific AI tools

The outlined challenges highlight a crucial need for domain-specific AI tools tailored to the unique demands of specialised fields, such as digital pathology. In transitioning towards these specialised solutions, the balance between cost and efficiency emerges as a key factor. 2023 advancements, such as FrugalGPT, show how to effectively harness LLMs in a cost-conscious way by utilising models tailored to specific queries.^12^ These tailored AI solutions leverage the extensive computational capabilities of AI, aligning them with the distinctive requirements of specialised domains. A case in point here is BioBERT, a derivative of the encoder representation with transformer (BERT) LLM. Although BERT was initially trained on general domain datasets (eg, English Wikipedia and Books Corpus), BioBERT underwent additional training using biomedical literature, including PubMed abstracts and PubMed Central full-text articles. This rigorous domain-specific training led to a substantial improvement in BioBERT’s performance in biomedical research tasks.^13^ Several techniques exist to boost the performance of LLMs or to reduce hallucination or inaccurate responses by integrating a domain-specific retrieval process into their operation. For instance, retrieval-augmented generation (RAG) works by dynamically retrieving relevant documents or data from a specialised database (eg, scientific literature that is specific to a particular specialty) at the time of the query, and then using these retrieved data to inform and augment the generation process of the LLM.^14^ Additionally, P-tuning is a technique that enhances the capabilities of LLMs by teaching them to recognise and exploit patterns within data to generate predictions or classifications. This method substantially improves upon traditional prompt tuning by introducing soft prompts that are learned through backpropagation and can be dynamically adjusted to optimise performance for specific tasks.^15–17^ By focusing on soft prompts learned through backpropagation, P-tuning adapts to different domains more robustly than hard-coded prompt methods, aiding in tasks, such as domain transfer and multi-task learning. Another popular method is reinforcement learning from human feedback, which allows for the iterative improvement of LLMs (and other AI systems) outputs based on human feedback, aligning the models’ behaviour with complex goals that are difficult to specify with traditional supervised learning techniques alone.^18^ The continuous evolution of such specialised tools signals a pathway towards increasingly precise and efficient solutions across various scientific disciplines (table 2).

### Domain-specific AI for digital pathology: GPT4DFCI-RAG

Recognising the immense potential of domain-specific generative AI in propelling medical research, our team undertook the rigorous task of curating a comprehensive digital pathology literature database. We initiated a targeted search on Google Scholar using specific keywords—“pathology”, “H&E”, “github”, “wsi”, and “machine learning”—to extract manuscripts, both peer-reviewed and preprints, dating from January, 2022 onwards. This search yielded 650 publications, capturing the latest developments in machine learning algorithms, innovative methodologies, new datasets, and applications in digital pathology. We then undertook a preprocessing phase, wherein we extracted the text from PDFs, cleaned the data, and augmented them with metadata to facilitate efficient integration into a curated, semantic search database.

Our approach combines GPT4DFCI, which is a private and secure generative AI tool based on GPT-4 Turbo deployed at Dana Farber Cancer Institute for non-clinical use,^27^ with a RAG architecture to create GPT4DFCI-RAG.^14^ We use RAG as an interface between the user and our curated database, without subjecting the model to further training or fine-tuning. Instead, we use RAG techniques to dynamically query the semantic database during a user interaction, allowing the model to generate accurate and contextually relevant responses based on the latest literature in digital pathology. We also require the model to provide evidence (ie, link to the specific publication and passage from which the information was derived) that supports each statement. This method ensures that the generative AI’s responses are directly informed by the content of the 650 PDFs, enhancing the specificity and relevance of the information provided to users. Importantly, this domain-specific generative AI tool operates under a closed world assumption, implying that knowledge of GPT4DFCI-RAG is confined to its curated corpus of PDFs. This design choice considerably reduces the chance of generating inaccurate or made-up responses—often referred to as hallucinations in AI jargon. With access to 14 404 pages of specialised literature, our tool echoes the ethos of Lilli by McKinsey^28^ and others detailed in table 2, albeit with a distinct focus on digital pathology.

To show the effectiveness of our web application we evaluated the performance of GPT4DFCI-RAG against the generic ChatGPT-4 model in responding to the same queries; both systems were powered by GPT-4 Turbo when compared. This evaluation leveraged a qualitative comparison of the accuracy of responses and the frequency of hallucinated responses generated by both models (appendix pp 1–7). We used a comprehensive set of queries designed to retrieve knowledge about the latest research findings in digital pathology (eg, the latest research papers about a particular task, such as predicting molecular alterations from whole slide images; state-of-the-art applications, such as visual-language foundation models for histopathology; or available histopathology imaging datasets with pixel-level annotations). Across the various examples, the responses generated by the digital pathology-specific tool (GPT4DFCI-RAG) were more relevant compared with those generated by the generalist ChatGPT-4 model (appendix pp 1–7). For instance, when responding to a query about the available histopathology imaging datasets with nuclei annotations, ChatGPT-4 listed non-relevant or inaccurate sources, such as The Cancer Genome Atlas and The Cancer Imaging Archive datasets, both of which are data archives that contain whole slide images without nuclear annotations (appendix p 6).^29,30^ ChatGPT-4 also showed a high rate of hallucinations, featuring papers or applications that do not exist, a phenomenon that was not observed with our digital pathology-specific model (appendix pp 1, 3, 5). Currently, although both GPT4DFCI-RAG and ChatGPT-4 were powered by GPT-4 Turbo, this comparison remains qualitative, pending the development of a ground-truth dataset that would enable more rigorous quantitative benchmarks. This assessment shows that domain-specific language models can overcome many of the limitations of general-purpose chatbots by providing accurate and precise responses with real references.

### Enhancing digital pathology analysis through PathML and ChatGPT-4 integration

In the landscape of computational pathology, the PathML library represents a major advancement, streamlining the analysis of vast and complex histopathology datasets.^31^ Developed with the guiding principles of scalability, standardisation, and ease of use, PathML facilitates the use of image analysis tools and AI algorithms in biomedical research. We have further augmented the utility of PathML by integrating its documentation with GPT-4, enabling users to interact with PathML through an intuitive chat interface, asking questions, and receiving guidance on the use of PathML for pathology image analysis.

This integration addresses a key need in the field of digital pathology: making advanced computational tools accessible to pathologists and wet laboratory scientists who might have little experience with programming. By interacting with the GPT-4-powered PathML chatbot, users can easily obtain accurate ready-to-use commands for common analysis tasks, such as tissue segmentation, biomarker quantification, and tumour classification (appendix pp 9–10). This interaction is not limited to command generation; users can also use this tool for explanations of PathML functions, best practices for histopathology data analysis, and troubleshooting advice, thus the chatbot is providing a comprehensive support system.

The practical application of PathML, coupled with ChatGPT-4 conversational AI capabilities, can be shown by several use cases that highlight the substantial reduction in time and effort required to perform complex analyses, with the added benefit of ChatGPT-4 guiding users through the process and enhancing their understanding of computational pathology (appendix pp 8–10). This seamless integration fosters a user-friendly environment that encourages the exploration and adoption of computational techniques in pathology. By making computational pathology more accessible, we not only enhance research capabilities but also pave the way for innovative applications by scientists whose little coding experience might constitute a barrier to contributing to the field.

## Potential implications: enhancing research efficiency and education

Although these AI tools themselves are not yet the agents of transformation in biomedical research and public health, these innovations can substantially increase productivity by streamlining access to knowledge and automating manual or repetitive tasks, thereby freeing up time for more crucial tasks and reducing the cognitive burden of keeping up with booming research topics.^32^ These tools can also lower the technical barrier for investigators who do not have specific skills, such as coding, empowering them to apply theoretical knowledge to their specific fields regardless of coding skills. In academic publishing, a notable example for this domain-specific language models includes tools, such as the Artificial Intelligence Review Assistant, developed by Frontiers (Lausanne, Switzerland) to automate the quality assessment of manuscripts and peer reviews and it is recommending editors and reviewers.^33^ Another notable application of these domain-specific tools is streamlining the process of conducting systematic reviews, a cornerstone in generating high-confidence evidence in medicine. Systematic reviews necessitate a thorough examination of existing literature to synthesise evidence on a particular research or clinical question, which traditionally demands a major investment of time and resources.^34^ In medical practice and education, UpToDate announced in 2023 their AI chatbot that uses their curated knowledge base and provides contextualised evidence.^35^ By automating the retrieval and preliminary analysis of relevant studies, domain-specific text-generative AI tools can drastically reduce the time required to do systematic reviews, enabling a more timely synthesis of evidence with an accuracy that supersedes that of LLMs.^36^

In public health, these tools can assist experts in consolidating findings from numerous epidemiological studies to identify effective interventions rapidly. A study by Jungwirth and colleagues^37^ discussed this very issue and reported that text-generative AI models can have major implications for public health by supporting research-driven decision making and developing new public health interventions, such as virtual health assistants. At the same time, the study acknowledges the limitations of LLMs including GPT-3 in fulfilling such implications and recommends fine-tuning to enhance their domain-specific performance and avoid amplifying the biases learned by the AI.^37^

Similarly, domain-specific text-generative AI tools are emerging as instrumental in advancing the fields of drug discovery and pharmaceutical research. Tools including ChemBERTa and ProtGPT2 (table 2) have been pre-trained on vast protein sequence datasets, acquiring an intricate grasp of protein structure and function. ProtGPT2, for instance, can generate de novo protein sequences,^25^ which could pave the way for new discoveries, while ChemBERTa has been fine-tuned to model chemical simplified molecular-input line entry system strings for toxicity prediction,^23^ which is a key aspect of drug development. Overall, the integration of these tools into the drug discovery process can possibly accelerate the transition from conceptual frameworks to practical applications.

## Conclusions

Domain-specific text-generative AI tools hold the potential to substantially enhance medical research and public health; however, the full potential of these domain-specific tools is still unfolding. Integrating these tools within academic and research frameworks could greatly enhance the efficiency and accuracy of knowledge retrieval from expanding scholarly databases, outperforming generalist LLMs. Additionally, as the cost of generalist LLM deployments continues to grow, smaller and specialised LLMs are poised to be more useful and cost-effective than their generalist counterparts; a likely future scenario we envision is the use of generalist LLMs (eg, ChatGPT) in the initial phases of research where ideas exploration and brainstorming take place and using smaller and specialised LLMs once a research problem is identified. Examples of the specialised LLMs are RAG architectures, such as the one we presented in this Viewpoint, possibly coupled with smaller models such as Microsoft Phi^38^ or Databricks Mosaic AI.^39^ Ultimately, as LLMs and RAG systems are advancing their capabilities in distilling and summarising complex textual information, having access to these tools in the research team promises to reduce manual workload. We will soon see how these systems allow researchers to increase effectiveness of scientific investigation.

## Supplementary Material

1

## Figures and Tables

**Table 1:** Overview of prominent large language models

	Developer	Key features	Training data size	Notable applications	
GPT-3^1^	OpenAI	175 billion parameters, autoregressive, transformer decoder architecture, and RLHF optimisation (GPT-3.5)	300 billion tokens	Contextual understanding, text generation, translation, and summarisation; the sub-class GPT-3.5 offers chat capability (GPT-3.5-turbo)	
GPT-4^2^	OpenAI	Estimated at about 1·8 trillion parameters, RLHF optimisation, and multimodal (text and images)	Not disclosed	Similar to GPT-3 and GPT-3.5, with the ability to process longer context, larger maximum token span, and the capacity to utilise multiple data modalities (text, images, etc)	
BERT^3^	Google	340 million parameters and bidirectional transformer encoder architecture	3·3 billion tokens	Sentiment analysis, text classification, and question answering	
T5^4^	Google	11 billion parameters, transformer architecture, and text-to-text transfer learning approach	1 trillion tokens	Text generation, translation, summarisation, and question answering	
LaMDA^5^	Google	137 billion parameters	768 billion tokens	Text generation and chat capability (included in Bard)	
Megatron-Turing NLG^6^	NVIDIA	530 billion parameters and large parallelism for training large models	338·6 billion tokens	Contextual understanding, text generation, natural language inference, and reading comprehension	
Llama 2^7^	Meta	70 billion parameters, RLHF, and IT optimisation	2 trillion tokens	Text generation, translation, and chat capability (Llama 2-Chat)	
PaLM^8^	Google	540 billion parameters	768 billion tokens	Text generation, translation, and chat capability (included in Bard)	
Gemini 1.5 Pro^9^	Google	Estimated at 1·5 trillion parameters and MoE transformer-based architecture	Estimated 30 trillion tokens	In-context learning, content analysis, summarisation, and classification; multimodal (text, images, audio, and video)	

BERT=Bidirectional Encoder Representation with Transformer. GPT=Generative Pre-trained Transformer. IT=instruction tuning. MoE=mixture-of-experts. NLG=natural language generation. RLHF=reinforcement learning from human feedback.

**Table 2:** Overview of notable domain-specific text-generation artificial inteligence tools in biomedicine

	Dataset	Key features
BioBERT^13^	PubMed abstracts, PubMed Central full texts	BERT derivative optimised for biomedical text mining
SciBERT^19^	Semantic Scholar’s corpus of 1·14 million papers	BERT derivative optimised for scientific text mining, classification, and named entity recognition
Med-BERT^20^	Electronic health records dataset of 28 490 650 patients from the Cerner database	BERT derivative optimised for structured electronic health records data
NYUTron^21^	7·25 million clinical notes from more than 300 000 patients within New York University Langone	BERT derivative optimised for medical language and fine-tuned for specific tasks including predicting inpatient mortality, comorbidity index, and readmission
GatorTron^22^	290 482 002 clinical notes from 2 476 628 patients at the University of Florida Health system together with PubMed articles and Wikipedia	BERT derivative optimised for clinical and biomedical text data and fine-tuned for five clinical natural language processing tasks: clinical concept extraction, medical relation extraction, semantic textual similarity, natural language inference, and medical question answering
ChemBERTa^23^	10 million SMILES from PubChem and ChEMBL	RoBERTa derivative optimised for chemical informatics, SMILES tokenisation
DNAGPT^24^	A dataset of 200 billion base pairs from all mammals	GPT derivative for DNA analysis including guanine–cytosine content prediction and sequence order prediction
ProtGPT2^25^	A dataset of 50 million non-annotated protein sequences	GPT derivative tailored for protein data, capable of generating de novo protein sequences
scGPT^26^	scRNA-seq profiles of 33 million normal human cells	GPT derivative tailored for scRNA-seq data, which can be used for cell type annotation, predicting unseen gene perturbations, and multi-batch and multi-omics integration

BERT=Bidirectional Encoder Representation with Transformer. RoBERTa=robustly optimised BERT approach. SMILES=simplified molecular-input line-entry system. GPT=Generative Pre-trained Transformer. scRNA-seq=single-cell RNA sequencing.
