# Supplementary material for: ChatGPT for digital pathology research
Source: Lancet Digit Health. Author manuscript; Available in PMC 2024 Aug 5. (PMC11299190; doi:10.1016/S2589-7500(24)00114-6)
Supplement: 1 [file NIHMS2012178-supplement-1.pdf]

THE LANCET
Digital Health

Supplementary appendix

This appendix formed part of the original submission and has been peer reviewed. We post it as supplied by the authors.

Supplement to: Omar M, Ullanat V, Loda M, Marchionni L, Umeton R. ChatGPT for digital pathology research. *Lancet Digit Health* 2024; published online July 9. https://doi.org/10.1016/ S2589-7500(24)00114-6.

Supplementary appendix

Table S1. Examples of the responses generated by our domain-specific digital pathology AI system (GPT4DFCI-RAG) and those generated by ChatGPT 4. Hallucinated responses, defined as completely made-up tool/paper, are highlighted in **red**. Wrong or irrelevant responses, defined as existing tool/paper but not directly related to the query, are highlighted in **orange**. Notably, (1) our AI system did not provide a single instance of hallucination or irrelevant information, (2) our AI system answers linked (as a superscript numbered reference, e.g.: [1]) the manuscripts where the information was sourced, (3) and both AI systems were based on GPT-4 Turbo.

Prompt: **List research papers that used deep learning algorithms on whole slide images to predict molecular alterations**

Ours (GPT4DFCI-RAG)	ChatGPT 4
The retrieved documents mention several research papers that have utilized deep learning algorithms on whole slide images (WSIs) for predicting molecular alterations. Here is a list of those papers:<br><br>1. "Development and validation of a weakly supervised deep learning framework to predict the status of molecular pathways and key mutations in colorectal" [1] .<br>2. "Deep learning identifies inflamed fat as a risk factor for lymph node metastasis in early colorectal cancer" [1] .<br>3. "Clinical-Grade Detection of Microsatellite Instability in Colorectal Tumors by Deep Learning" [1] .<br>4. "Deep learning for the detection of microsatellite instability from histology images in colorectal cancer: A systematic literature review" [1] .<br>5. "Deep learning identifies morphological features in breast cancer predictive of cancer ERBB2 status and trastuzumab treatment efficacy" [1] .<br>6. "Deep learning links histology, molecular signatures and prognosis in cancer" [1] .<br>7. "Classification and mutation prediction from non-small cell lung cancer histopathology images using deep learning" [1] .<br>8. "Deep learning in cancer pathology: a new generation of clinical biomarkers" [1] .<br>9. "Deepsmile: Contrastive self-supervised pretraining benefits MSI and HRD classification directly from H&E whole-slide images in colorectal and breast cancer" [2] .<br>10. "Deep biological pathway informed pathology-genomic multimodal survival prediction" [2] .<br>11. "Deep learning-based integration of histology, radiology, and genomics for improved survival prediction in glioma patients" [2] .<br>12. "Predicting cancer outcomes from histology and genomics using convolutional networks" .<br><br>Please note that the list above is based on the information provided in the retrieved documents and may not be exhaustive of all research papers in the field.	Compiling a comprehensive and up-to-date list of all research papers that utilize deep learning algorithms on whole slide images (WSIs) for predicting molecular alterations is a complex task that requires access to multiple scientific databases and continuous monitoring of new publications. However, I can highlight several key studies in this domain to give you an idea of the landscape. This list will include some seminal works and recent impactful papers, but it is by no means exhaustive. For the most current research, I recommend searching databases like PubMed, Google Scholar, and arXiv.<br><br>Seminal and Recent Impactful Papers:<br>**1. "Deep learning-based transformation of H&E stained tissues into special stains" by Bayramoglu, N., & Heikkilä, J. (2016). This paper discusses the use of deep learning for transforming H&E stained tissue images into images that resemble those stained with other chemicals, indicating an early exploration of deep learning in histopathology.**<br>**2. "Predicting cancer outcomes from histology and genomics using convolutional networks" by Coudray, N., Ocampo, P.S., Sakellaropoulos, T., Narula, N., Snuderl, M., Fenyö, D., Moreira, A.L., Razavian, N., & Tsirigos, A. (2018). In this study, deep convolutional neural networks were used to predict gene mutations in lung cancer from WSIs, demonstrating the potential of deep learning in predicting molecular alterations directly from histology.**<br>**3. "Artificial intelligence in digital pathology — new tools for diagnosis and precision oncology" by Pantanowitz, L., & Parwani, A. (2019). While not a primary research paper, this review discusses the impact of artificial intelligence, including deep learning on digital pathology, highlighting advancements and potential applications in predicting molecular alterations.**<br>**4. "AI-based pathology predicts origins for cancers of unknown primary" by Kather, J.N., Pearson, A.T., Halama, N., Jäger, D., Krause, J., Loosen, S.H., Marx, A., Boor, P., Tacke, F., Neumann, U.P., Grabsch, H.I., Yoshikawa, T., Brenner, H., Chang-Claude, J., Hoffmeister, M., Trautwein, C., & Luedde, T. (2020). This research demonstrates the use of AI in accurately predicting the primary sites of metastatic cancers from WSIs, showing the utility of deep learning in understanding complex molecular backgrounds.**<br>**5. "Integrative Analysis of Pathology Images and Genomic Data Predicts Survival in Breast Cancer Patients" by Mobadersany, P., Yousefi, S., Amgad, M., Gutman, D.A., Barnholtz-Sloan, J.S., Vega, J.E.V., Brat, D.J., & Cooper, L.A.D. (2018). This study illustrates how integrating deep learning-based analysis of WSIs with genomic data can improve survival prediction in breast cancer patients, indicating a move towards more holistic approaches in cancer prognosis.**<br><br>For the latest papers, you should use specific search queries related to your field of interest, like "deep learning whole slide images molecular alterations" in scientific databases. Furthermore, tools like Google Scholar's alert function can help you stay up-to-date with newly published research in this rapidly evolving field.

Prompt: **What molecular alterations were the focus of the study titled "Development and validation of a weakly supervised deep learning framework to predict the status of molecular pathways and key mutations in colorectal cancer from routine histology images"**

Ours (GPT4DFCI-RAG)	ChatGPT 4
The study titled "Development and validation of a weakly supervised deep learning framework to predict the status of molecular pathways and key mutations in colorectal cancer from routine histology images" focused on predicting the status of molecular pathways and key mutations in colorectal cancer. The specific molecular alterations that were the focus of the study included microsatellite instability (MSI), hypermutation density, chromosomal instability, CpG island methylator phenotype (CIMP) high prediction, BRAF mutation, and TP53 mutation [1]. These molecular features are significant as they can influence the prognosis and treatment strategies for colorectal cancer patients.	The study titled "Development and validation of a weakly supervised deep learning framework to predict the status of molecular pathways and key mutations in colorectal cancer from routine histology images" primarily focused on leveraging deep learning to predict the status of key molecular pathways and specific mutations in colorectal cancer (CRC) based on routine hematoxylin and eosin (H&E) stained histology images. Although I cannot provide specific details from the study without accessing it directly, typically, studies like this aim to predict alterations in several key molecular pathways and mutations known to be significant in the pathogenesis and treatment response of colorectal cancer. Common targets for such studies include:<br>1. APC Mutation: A common mutation in CRC, involved in the Wnt signaling pathway, which plays a crucial role in cell cycle regulation and apoptosis.<br>2. KRAS Mutation: KRAS mutations are significant for CRC as they can influence the effectiveness of EGFR-targeted therapies.<br>3. BRAF Mutation: The BRAF V600E mutation is another critical mutation in CRC, associated with a poor prognosis and resistance to some therapies.<br>4. Microsatellite Instability (MSI): High MSI status is a marker for Lynch syndrome and has implications for prognosis and immunotherapy response.<br>5. TP53 Mutation: Mutations in TP53 are common in many cancers, including CRC, and are involved in cell cycle control, apoptosis, and DNA repair.<br>The study likely used a weakly supervised learning approach because obtaining large datasets with detailed annotations of molecular pathway statuses and specific mutations from histology images can be challenging. Instead, this approach allows the model to learn from less-specific labels, such as the overall presence or absence of certain mutations or pathway activations inferred from clinical or genomic data, to make predictions directly from the histology images.<br>For precise information about which molecular alterations were the focus of the study and how the deep learning framework was developed and validated, the original article would need to be consulted. Researchers in digital pathology and computational biology, such as yourself, might find this study's methodology and findings particularly relevant for advancing the field of predictive modeling in oncology.

Prompt: **List research papers that used deep learning models for deriving Gleason score from WSIs of prostate cancer**

Ours (GPT4DFCI-RAG)

The retrieved documents mention several research papers that have utilized deep learning models for deriving Gleason scores from whole slide images (WSIs) of prostate cancer. Here is a list of those papers:

1. "A Pathologist-Informed Workflow for Classification of Prostate Glands in Histopathology" by Alessandro Ferrero, Beatrice Knudsen, Deepika Sirohi, and Ross Whitaker [1] .
2. "Artificial intelligence for diagnosis and Gleason grading of prostate cancer: The PANDA challenge" [2] .
3. "A deep learning system for prostate cancer diagnosis and grading in whole slide images of core needle biopsies" [2] .
4. "Computer aided analysis of prostate histopathology images to support a refined Gleason grading system" [2] .
5. "Automated deep-learning system for Gleason grading of prostate cancer using biopsies: A diagnostic study" [2] .
6. "Deep learning for automatic Gleason pattern classification for grade group determination of prostate biopsies" [2] .
7. "Convolutional neural networks for an automatic classification of prostate tissue slides with high-grade Gleason score" [2] .
8. "Development and validation of a deep learning algorithm for improving Gleason scoring of prostate cancer" [2] .
9. "Deep Learning in Prostate Cancer Diagnosis and Gleason Grading in Histopathology Images: An Extensive Study" [3] .
10. "Machine learning approaches to analyze histological images of tissues from radical prostatectomies" [3] .
11. "Automatic gleason grading of prostate cancer using quantitative phase imaging and machine learning" [3] .
12. "Detecting prostate cancer using deep learning convolution neural network with transfer learning approach" [3] .
13. "Automated gleason grading of prostate cancer tissue microarrays via deep learning" [3] .
14. "High-accuracy prostate cancer pathology using deep learning" [3] .
15. "Development and validation of a deep learning algorithm for improving gleason scoring of prostate cancer" [3] .
16. "Deep learning for digital pathology image analysis: A comprehensive tutorial with selected use cases" [3] .
17. "Deep learning in cancer pathology: a new generation of clinical biomarkers" [3] .
18. "Artificial intelligence for diagnosis and grading of prostate cancer in biopsies: a population-based, diagnostic study" [3] .
19. "Artificial intelligence for diagnosis and Gleason grading of prostate cancer: the PANDA challenge" [3] .
20. "Going deeper through the gleason scoring scale: An automatic end-to-end system for histology prostate grading and cribriform pattern detection" [3] .
21. "Deep learning for automatic gleason pattern classification for grade group determination of prostate biopsies" [3] .
22. "Development and validation of an artificial intelligence-powered platform for prostate cancer grading and quantification" [3] .
23. "Deep learning-based gleason grading of prostate cancer from histopathology images--role of multiscale decision aggregation and data augmentation" [4] .
24. "Multiple Instance Learning with Mixed Supervision in Gleason Grading" by Hao Bian, Zhuchen Shao, Yang Chen, Yifeng Wang, Haoqian Wang, Jian Zhang, and Yongbing Zhang [5] .

These papers represent a selection of the research conducted in the field of digital pathology, specifically using deep learning for the Gleason grading of prostate cancer from WSIs.

ChatGPT 4

Deep learning models have been increasingly applied to Whole Slide Images (WSIs) of prostate cancer for various diagnostic and prognostic purposes, including the derivation of the Gleason score. The Gleason score is a critical factor in prostate cancer grading, providing insights into the aggressiveness of the cancer based on the histological patterns observed in prostate tissue. Here is a list of research papers that have utilized deep learning models to derive Gleason scores from WSIs:

1. "Development and Validation of a Deep Learning Algorithm for Improving Gleason Scoring of Prostate Cancer" - This study by Campanella et al. demonstrates the use of a deep learning algorithm to automate and improve the accuracy of Gleason scoring in prostate cancer WSIs. [NPJ Digital Medicine, 2019]
2. "Deep Learning for Automatic Gleason Pattern Classification for Grade Group Determination of Prostate Biopsies" - Bulten et al. presented a deep learning model capable of classifying Gleason patterns in prostate biopsies, thereby aiding in the accurate determination of grade groups. [Virchows Archiv, 2019]
3. "Artificial Intelligence for Diagnosis and Grading of Prostate Cancer in Biopsies: A Population-Based, Diagnostic Study" - This study by Ström et al. explores the use of artificial intelligence, specifically deep learning, in diagnosing and grading prostate cancer in biopsy samples, including Gleason score determination. [The Lancet Oncology, 2020]
4. "Automated Gleason Grading of Prostate Cancer Tissue Microarrays via Deep Learning" - Nagpal et al. developed a deep learning approach to automate the Gleason grading process in prostate cancer tissue microarrays, showcasing the potential for enhancing diagnostic workflows. [Scientific Reports, 2019]
5. **"Deep Learning-Assisted Diagnosis of Prostate Cancer with Gleason Score Prediction on Biopsy Slides" - This paper by Lucas et al. focuses on the application of deep learning models to assist in the diagnosis of prostate cancer and predict Gleason scores from biopsy slides, highlighting the model's diagnostic performance. [IEEE Access, 2019]**
6. **"A Deep Learning Model to Predict a Diagnosis of Prostate Cancer in Whole-Slide Images of Biopsies" - Pantanowitz et al. investigated the feasibility of using deep learning models to predict prostate cancer diagnosis and provide an estimation of the Gleason score directly from WSIs of prostate biopsies. [JAMA Oncology, 2020]**

These studies represent significant efforts in integrating deep learning technologies with pathology to improve the accuracy and efficiency of prostate cancer diagnosis and grading. They underscore the potential of computational approaches, such as deep learning, to augment traditional pathology methods, offering more precise and reproducible assessments of critical factors like the Gleason score.

Prompt: **List the available visual-language foundation models available for histopathology WSIs?**

Ours (GPT4DFCI-RAG)	ChatGPT 4
The available visual-language foundation models for histopathology Whole Slide Images (WSIs) mentioned in the retrieved documents include:<br><br>1. CONtrastive learning from Captions for Histopathology (CONCH) - A visual-language foundation model developed using diverse sources of histopathology images, biomedical text, and over 1.17 million image-caption pairs via task-agnostic pretraining [1] .<br>2. PLIP - A visual-language foundation model mentioned as a concurrent system to CONCH [2] .<br>3. BiomedCLIP - Another visual-language foundation model that is compared with CONCH in the documents [2] .<br>4. OpenAICLIP - A general visual-language model that has been applied to histopathology and is referenced in the documents [2] .<br><br>These models are designed to be transferred to a wide range of downstream tasks involving either or both histopathology images and text, achieving state-of-the-art performance on various histology image classification, segmentation, captioning, text-to-image, and image-to-text retrieval tasks.	Visual-language foundation models designed for analyzing histopathology whole slide images (WSIs) represent a growing area of interest in digital pathology, leveraging the advances in AI to interpret complex biological data. Here are some notable models and approaches that integrate visual (image) and language (textual annotation or description) data for the analysis of histopathology images:<br><br>**1. CLIP (Contrastive Language–Image Pre-training):**<br>• **While CLIP itself is not specifically tailored for histopathology, its architecture, which jointly learns from images and text through a contrastive loss, has inspired adaptations in the medical imaging field. For instance, researchers can train modified versions of CLIP on paired images and textual pathology reports to improve the model's ability to understand and annotate histopathological features.**<br>**2. BioViL (Biomedical Vision-Language):**<br>• **BioViL is an adaptation of the vision-language model specifically for biomedical contexts. It can be pre-trained on a large dataset of medical images and corresponding annotations, then fine-tuned for specific tasks like disease detection or progression in histopathology slides.**<br>3. Pathomic Fusion:<br>• This model focuses on integrating multimodal data, including genetic information (omic data) and histopathology images, using a fusion approach to predict clinical outcomes more accurately. While not strictly a visual-language model, it exemplifies the integration of different types of biological data, akin to combining image and text.<br>**4. Domain-specific adaptations of BERT (Bidirectional Encoder Representations from Transformers) for pathology reports:**<br>• **BERT and other transformer-based models have been adapted to process pathology reports. These models can be combined with image analysis models (like CNNs) in a dual-stream architecture to enhance performance on tasks like tumor classification or mutation prediction from WSIs.**<br>**5. Custom Transformer Models:**<br>• **Researchers have started to develop transformer-based models that directly incorporate both histopathology images and associated textual data (like clinical notes or pathology reports) to perform tasks such as diagnosis prediction, prognosis estimation, or even gene expression profiling.**<br><br>These models typically require substantial datasets with well-annotated images and corresponding text data for training. The success of these systems depends heavily on the quality and quantity of the dataset, as well as the specificity of the annotations provided. In your case, developing or adapting a visual-language model could involve training on datasets comprising WSIs and detailed annotations describing the histopathological features and clinical information pertinent to prostate cancer.

Prompt: **How did CONCH compare to the other visual-language foundation models in terms of accuracy?**

Ours (GPT4DFCI-RAG)	ChatGPT 4
CONCH (CONtrastive learning from Captions for Histopathology) outperformed other visual-language foundation models in terms of accuracy across various tasks. On slide-level tasks, CONCH achieved a balanced accuracy score of 84.7%, and an average accuracy of 90.5% across three tasks, which was higher than PLIP and BiomedCLIP, which had 86.6% and 87.9% respectively, although no statistical significance was detected for each task [1] .<br><br>In the ROI-level tasks, CONCH performed nearly identically to the state-of-the-art CTransPath encoder, with 93.0% vs. 93.8% balanced accuracy on CRC100k and 0.846 vs. 0.835 quadratic weighted Cohen's kappa on SICAP. It also outperformed PLIP, BiomedCLIP, and OpenAICLIP by between 3.4% and 5.1% in balanced accuracy on CRC100k and between 0.084 and 0.142 in quadratic weighted Cohen's kappa on SICAP ($p < 0.01$ for all comparisons) [1] .<br><br>For instance, in BRCA subtyping, using the CONCH encoder with 8 training labels per class outperformed using PLIP, BiomedCLIP, or OpenAICLIP with 64 labels per class, representing an 8-fold reduction in training set size. This trend was also observed for most tasks tested [1] . Additionally, CONCH's zero-shot performance was highly competitive when compared to few-shot supervised learning, often requiring fewer labels to achieve the same performance [1] .<br><br>On slide-level benchmarks, CONCH outperformed state-of-the-art visual-language foundation models (PLIP, BiomedCLIP, and OpenAICLIP) on all tasks, often by a wide margin. For example, for NSCLC subtyping and RCC subtyping, CONCH achieved zero-shot accuracies of 90.0% and 89.3% respectively, outperforming the next best performing model, PLIP, by 11.3% and 8.9% on each task with $p < 0.01$ [2] .<br><br>In more challenging tasks like invasive breast carcinoma (BRCA) subtyping and LUAD pattern classification, CONCH achieved zero-shot accuracies of 84.0% and a Cohen's kappa score of 0.236, significantly higher than other models [2] .<br><br>Overall, these results demonstrate that CONCH provides a strong image encoder that either performs comparably to or better than all visual encoders tested, including the self-supervised state-of-the-art method [1]	**The CONCH model, which stands for "CONtrastive learning for Compositional Hypernymy," was introduced in a research paper titled "CONCH: Compositional Hypernymy with Contrastive Learning" in 2021. The primary focus of CONCH is on improving the understanding of compositional hypernymy (a type of word relation where the meaning of one word encompasses the meaning of another, like "animal" and "dog") using contrastive learning techniques. This model is particularly aimed at natural language processing (NLP) tasks related to word relationships and semantic composition.**<br><br>**CONCH was not specifically compared against visual-language foundation models since it's more focused on textual semantic relationships rather than multimodal (visual and language) tasks. Visual-language foundation models, such as CLIP by OpenAI or Google's ALIGN, are designed to understand and correlate information across both visual and textual data, which is a broader scope compared to what CONCH aims to address.**

Prompt: **list all available histopathology slide datasets with nuclei annotation**

Ours (GPT4DFCI-RAG)

The retrieved documents mention several datasets that include nuclei annotation. Here is a list of the datasets mentioned:

1. TUPAC16 - This dataset contains 73 whole slide images (WSIs) in PNG format, annotated with text files containing the coordinates of mitosis figures [1] .
2. JPATHOL - This dataset is prepared for experimentation with nuclei, epithelium, and tubule segmentation tasks. It is comprised of three separate medical datasets [1] .
3. NuCLS - A dataset for nucleus classification, localization, and segmentation [2] .
4. Lizard - A large-scale dataset for colonic nuclear instance segmentation and classification [2] .
5. CoNIC - Colon nuclei identification and counting challenge 2022 [2] .
6. BreCaHAD - A real-world dataset with 176 image tiles and 23,549 annotated nuclei [3] .
7. PanNuke - A real-world dataset with 7,901 image tiles and 216,400 annotated nuclei [3] .
8. TNBC - A real-world dataset with 50 image tiles and 4,022 annotated nuclei [3] .
9. Synthetic Nuclei and annOtation Wizard (SNOW) - A large-scale synthetic pathological image dataset paired with annotation for nuclei semantic segmentation [3] .
10. CoNSeP - A dataset mentioned in the context of the difficulties of labeling nuclei in histopathological images [4] .
11. Lizard (mentioned again in a different context) - The largest publicly available dataset for nuclear segmentation and classification in computational pathology [4] .

Please note that some datasets are mentioned in the context of the challenges associated with nuclei annotation and may not be primarily focused on nuclei annotation but include it as part of their data.

ChatGPT 4

When it comes to datasets with nuclei annotations, particularly useful for tasks in digital pathology and computational biology, several notable ones are available across various platforms. Here's a list of some widely-used datasets:

1. **The Cancer Genome Atlas (TCGA): While primarily known for its genomic data, TCGA also includes histopathological images (H&E stained) that often come with expert annotations, including nuclei.**
2. **The Cancer Imaging Archive (TCIA): Similar to TCGA, TCIA offers a variety of imaging data, including annotated histological images which can be useful for nuclei studies.**
3. MoNuSeg (Multi-organ Nuclei Segmentation Challenge): Specifically designed for nuclei segmentation, this dataset includes annotated nuclei from multiple organs, derived from both TCGA and TCIA.
4. The Kumar dataset: Released as part of a challenge for nuclei segmentation, it includes images from multiple tissue types with detailed nuclei annotations.
5. **GlaS@MICCAI2015 (Gland Segmentation in Colon Histology Images): Though focused on gland segmentation, this dataset includes detailed structure annotations that can indirectly aid in nuclei annotation studies.**
6. **Lung and Colon Cancer Histopathological Images (LC25000): This dataset includes labeled images of lung and colon cancers, where nuclei annotations can be indirectly inferred through cancer cell annotations.**
7. **BraTS (Brain Tumor Segmentation Challenge): While primarily brain tumor images, some datasets include detailed annotations that can help with nuclei studies, especially in tumor regions.**

These datasets are often used in research for developing and benchmarking algorithms for tasks like nuclei detection, segmentation, and classification in various tissue types. Each dataset comes with its own set of rules and accessibility options, so it's important to check their respective websites for usage guidelines and licensing information.

Prompt: **Give a brief description of the PANDA challenge dataset**

Ours (GPT4DFCI-RAG)	ChatGPT 4
The PANDA challenge dataset is a large collection of whole slide images (WSIs) used for the development of deep learning-based systems, particularly for the grading of prostate cancer using the International Society of Urological Pathology (ISUP) grading system. A total of 10,616 WSIs were used in the study, which are available from the Prostate cANcer graDe Assessment (PANDA) challenge. The dataset includes WSIs collected from Radboud University Medical Center (5160 WSIs) and Karolinska Institute (5456 WSIs), with a distribution of ISUP scores from 0 to 5 across the samples [1] .<br><br>The PANDA challenge aimed to use this large dataset to develop a deep-learning-based ISUP grading system, evaluate the generalizability of the automated ISUP grading, and incorporate label distribution learning (LDL) in system development to address differences in label characteristics between datasets [1] . The dataset was split into a development set (training/validation sets) and an unseen test set, with the development set consisting of WSIs obtained from the two institutions mentioned above [1] .<br><br>The dataset was used in the PANDA challenge, which was a global AI competition organized to catalyze the development of reproducible AI algorithms for Gleason grading of digitized prostate biopsies. The challenge was the largest histopathology competition to date, joined by 1,290 developers, and it was the largest publicly available dataset of prostate biopsies at the time [2] .<br><br>The PANDA challenge dataset is publicly available for further research, and the most up-to-date information regarding the dataset can be found on the challenge website. Additionally, code used to generate the results of the various algorithms and example code on how to load the images in the PANDA dataset is available online [2] [3] .	The PANDA (Prostate cANcer graDe Assessment) Challenge dataset is a collection of digitized whole-slide images of prostate biopsies used for a competition hosted on Kaggle. This dataset was developed in collaboration between the Karolinska Institute in Sweden, Radboud University Medical Center in the Netherlands, and Google Health. The primary objective of the challenge was to improve the accuracy of Gleason scoring, which is a grading system used to determine the aggressiveness of prostate cancer.<br><br>The dataset includes whole-slide images (WSIs) stained with Hematoxylin and Eosin (H&E), labeled with both the Gleason pattern and the ISUP grade, which is a more recent grading system correlated with the Gleason score but designed to be more consistent. Each slide is annotated by experts, providing valuable training data for developing machine learning models aimed at automating the grading process.<br><br>The PANDA Challenge specifically aimed to encourage the development of algorithms that can accurately predict the ISUP grade based on these WSIs, potentially leading to more consistent and reproducible diagnosis of prostate cancer. The dataset is large and comprehensive, making it suitable for training sophisticated computational models like deep learning for tasks in digital pathology.

RAG: Retrieval-Augmented Generation

Table S2. Examples of usage of our GPT4-powered PathML Chatbot, designed to provide code and instructions on using PathML for image analysis in response to text prompts.

Prompt: **How do I install PathML on a mac?**

To install PathML on a Mac, you will follow these steps. Note that this process involves using the terminal and requires that you have Conda installed. If you do not have Conda, you can download Miniconda here. Here are the detailed steps:

1. Create a Conda environment: Open your terminal and create a new Conda environment specifically for PathML. This environment will isolate the PathML dependencies from other Python packages you may have installed. You can create the environment with Python 3.8 (recommended for compatibility) by running:

```
conda create --name pathml python=3.8
```

2. Activate the Conda environment: Once the environment is created, you need to activate it. You can do this by running:

```
conda activate pathml
```

3. Install external dependencies: Before installing PathML, you must install its external dependencies. On a Mac, this primarily involves installing OpenSlide. You can do this using Homebrew, a package manager for macOS. If you do not have Homebrew installed, you can find installation instructions on the Homebrew website. Install OpenSlide by running:

```
brew install openslide
```

4. Install OpenJDK 8: PathML requires Java, specifically OpenJDK version 8. You can install it within your Conda environment by running:

```
conda install openjdk==8.0.152
```

5. Install PathML: Finally, with all dependencies in place, you can install PathML using pip. Run the following command to install it:

```
pip install pathml
```

After completing these steps, PathML should be installed in your Conda environment. You can start using it for your digital pathology analysis projects.

Prompt: **How can I create an imaging processing transform that is compatible with PathML?**

To create an imaging processing transform that is compatible with PathML, you'll want to define a custom transform class that inherits from PathML's base transform class. This involves implementing the __call__ method, where you define the processing logic for your custom transform. Below is a general template to guide you through creating a custom imaging processing transform in PathML:

```
from pathml.preprocessing.transforms import Transform

class CustomTransform(Transform):
    def __init__(self, *args, **kwargs):
        # Initialize any parameters your transform needs here
        super().__init__()

    def __call__(self, slide):
        # Implement your transform logic here
        # The slide parameter is a SlideData object

        # Example processing logic:
        # This could be anything from filtering, segmentation, normalization, etc.
        # You can access the image data using slide.slide, and you should modify this array
        # or create a new one based on your processing needs

        # After processing, make sure to update the slide object as needed

        # For example, if you create a new processed image, you might do:
        # slide.slide = processed_image

        # Return the modified slide object
        return slide
```

In this template, CustomTransform is your new transform class that applies your specific imaging processing algorithm to a SlideData object. The __call__ method is where the actual processing happens, and it must take a SlideData object as input and return the modified SlideData object. Here's a more concrete example, demonstrating a hypothetical transform that applies a simple Gaussian blur to the image data:

```
import cv2
import numpy as np
from pathml.preprocessing.transforms import Transform

class GaussianBlurTransform(Transform):
    def __init__(self, kernel_size=(5, 5), sigmaX=0):
        super().__init__()
        self.kernel_size = kernel_size
        self.sigmaX = sigmaX

    def __call__(self, slide):
        # Assuming slide.slide is a numpy array of the image
        # Apply Gaussian Blur to each tile in the slide
        for tile in slide.tiles.values():
            tile.image = cv2.GaussianBlur(tile.image, self.kernel_size, self.sigmaX)

        return slide
```

This GaussianBlurTransform class applies a Gaussian blur to each tile's image in the slide. You can customize the kernel size and sigmaX value for the blur through the class constructor. To use this custom transform in a PathML pipeline, you would do something like this:

```
from pathml.core import HESlide
from pathml.preprocessing import Pipeline

# Initialize your custom transform with any parameters it needs
gaussian_blur_transform = GaussianBlurTransform(kernel_size=(5, 5), sigmaX=1)

# Create a pipeline with your custom transform
pipeline = Pipeline([gaussian_blur_transform])

# Load a slide
slide = HESlide(filepath="path/to/your/slide.svs", name="ExampleSlide")

# Run the pipeline on your slide
processed_slide = slide.run(pipeline)
```

Prompt: **How do I quantify the intensity of a nuclear marker in a Vectra Polaris image?**

To quantify the intensity of a nuclear marker in a Vectra Polaris image using the PathML library, you need to follow these steps:

1. Load the Vectra Polaris Image: Use the VectraSlide class to load the image.

2. Preprocess the Image: Apply necessary preprocessing steps such as CollapseRunsVectra to collapse multiple runs into a single representative run for analysis.

3. Segment the Image: Use the SegmentMIF transform for segmenting the image. Even though Vectra Polaris images are multiplex immunofluorescence images, the segmentation step requires identifying the nuclear regions which SegmentMIF can handle given it's designed for multiplex IF images.

4. Quantify Marker Intensity: After segmentation, use QuantifyMIF to quantify the intensity of the nuclear marker within the segmented regions.

Here is an example code snippet that demonstrates these steps:

```
from pathml.core import VectraSlide
from pathml.preprocessing import Pipeline
from pathml.preprocessing.transforms import CollapseRunsVectra, SegmentMIF, QuantifyMIF

# Step 1: Load the Vectra Polaris Image
slide_path = "/path/to/your/vectra/polaris/image.tiff"
vectra_slide = VectraSlide(filepath=slide_path)

# Step 2: Preprocess the Image
# Collapse multiple runs (if necessary)
preprocessing_pipeline = Pipeline([
    CollapseRunsVectra()
])

vectra_slide.run(preprocessing_pipeline)

# Step 3: Segment the Image
# Assuming nuclear_channel corresponds to the index of the nuclear marker
# Adjust 'nuclear_channel' based on your specific image
segmentation_pipeline = Pipeline([
    SegmentMIF(model="mesmer", nuclear_channel=0, image_resolution=0.5)
])

vectra_slide.run(segmentation_pipeline)

# Step 4: Quantify Marker Intensity
# Quantify the intensity of the nuclear marker
quantification_pipeline = Pipeline([
    QuantifyMIF(segmentation_mask="nuclear_segmentation")
])

vectra_slide.run(quantification_pipeline)

# The results of the quantification will be stored in vectra_slide, which can be accessed as needed
```
